# Persistent immune imprinting after XBB.1.5 COVID vaccination in humans

**DOI:** 10.1101/2023.11.28.569129

**Published:** 2023-11-30

**Authors:** M. Alejandra Tortorici, Amin Addetia, Albert J. Seo, Jack Brown, Kaitlin R. Sprouse, Jenni Logue, Erica Clark, Nicholas Franko, Helen Chu, David Veesler

**Affiliations:** 1 Department of Biochemistry, University of Washington, Seattle, WA, USA; 2 Division of Allergy and Infectious Diseases, University of Washington, Seattle, WA, USA; 3 Howard Hughes Medical Institute, University of Washington, Seattle, WA, USA

## Abstract

Immune imprinting - also known as ‘original antigenic sin’ - describes how the first exposure to a virus shapes the immunological outcome of subsequent exposures to antigenically related strains. SARS-CoV-2 Omicron breakthrough infections and bivalent COVID-19 vaccination were shown to primarily recall cross-reactive memory B cells and antibodies induced by prior mRNA vaccination with the Wuhan-Hu-1 spike rather than priming naive B cells that recognize Omicron-specific epitopes. These findings underscored a strong immune imprinting resulting from repeated Wuhan-Hu-1 spike exposures. To understand if immune imprinting can be overcome, we investigated memory and plasma antibody responses after administration of the updated XBB.1.5 COVID mRNA vaccine booster. Our data show that the XBB.1.5 booster elicits neutralizing antibody responses against current variants that are dominated by recall of pre-existing memory B cells previously induced by the Wuhan-Hu-1 spike. These results indicate that immune imprinting persists even after multiple exposures to Omicron spikes through vaccination and infection, including post XBB.1.5 spike booster mRNA vaccination, which will need to be considered to guide the design of future vaccine boosters.

Emergence of immune evasive SARS-CoV-2 variants erodes the effectiveness of COVID-19 vaccines, which led to the development of two updated vaccine boosters. A bivalent Wuhan-Hu-1/BA.5 (or BA.1 for a few countries) spike (S) mRNA booster vaccine was approved in August 2022^1,2^ and, subsequently, a monovalent XBB.1.5 S mRNA booster vaccine in September 2023^3^. Neutralizing antibodies are a correlate of protection against COVID-19^4–7^ and most plasma neutralizing activity is directed to the S receptor-binding domain (RBD)^8–10^. Recent studies showed that antibody responses to Omicron variants are dominated by pre-existing immunity resulting from prior exposure to the Wuhan-Hu-1 spike , due to immune imprinting^11–15^. A study of individuals receiving inactivated Wuhan-Hu-1 viral vaccines, however, found that repeated Omicron infections could overcome immune imprinting, leading to elicitation of *de novo* antibody responses specific for these variants^16^. It is unknown if a similar outcome can be achieved through repeated administration of updated vaccine boosters in individuals previously imprinted via multiple Wuhan-Hu-1 S exposure.

To evaluate humoral immunity elicited upon receipt of an XBB.1.5 S mRNA vaccine booster, we collected plasma from individuals who had previously received multiple vaccine doses with or without known infection (Tables S1–S2 in the Supplementary Appendix). We used a vesicular stomatitis virus (VSV) pseudotyped with the Wuhan-Hu-1/D614G S, BQ.1.1 S, XBB.1.5 S or the BA.2.86 S to assess the potency and breadth of plasma neutralizing antibodies in this cohort and compare them with plasma collected upon receipt of the bivalent Wuhan-Hu-1/BA.5 vaccine booster. Neutralizing activity was highest against the Wuhan-Hu-1/G614 S VSV with geometric mean titers (GMTs) of 1,700 and 3,000 after receiving the XBB.1.5 S and the Wuhan-Hu-1/BA.5 S bivalent vaccine boosters, respectively. GMTs against BQ.1.1, XBB.1.5 and BA.2.86 S VSV were 510, 540 and 480 (after XBB.1.5 S vaccination) or 420, 240 and 320 (after bivalent vaccination), respectively. (Figure 1A–B, Figure S1 in the Supplementary Appendix). Our data show that the updated vaccine booster elicits neutralizing antibody responses against current variants, which is expected to translate into enhanced protection in the real world.

The finding that administration of an XBB.1.5 S booster elicited higher plasma neutralizing activity against Wuhan-Hu-1/D614G S VSV (vaccine-mismatched) relative to XBB.1.5 S VSV (vaccine-matched) is a serological indication of immune imprinting. To investigate this further, we depleted polyclonal plasma antibodies recognizing the Wuhan-Hu-1 S trimer and assessed binding titers against the Wuhan-Hu-1 S and XBB.1.5 S ectodomain trimers using ELISAs. As expected, no antibodies binding to Wuhan-Hu-1 S were detected after depletion. Moreover, depletion of antibodies targeting Wuhan-Hu-1 S entirely abrogated binding to XBB.1.5 S except for two individuals for which binding titers decreased 10-fold (Figure 1C–D, Figure S2 in the Supplementary Appendix). Accordingly, we did not detect neutralizing antibodies against Wuhan-Hu-1/D614G S VSV and XBB.1.5 S VSV following depletion (Figure 1E–F, Figure S3 in the Supplementary Appendix), indicating the absence of XBB.1.5 S-specific antibodies in the plasma of these subjects (i.e. that were not cross-reactive with Wuhan-Hu-1 S). These data suggest that XBB.1.5 S vaccination increased cross-reactive plasma antibody titers previously elicited by Wuhan-Hu-1 S exposure, which are also binding to and neutralizing XBB.1.5 and other variants, instead of inducing *de novo* antibody responses against XBB.1.5 S.

We subsequently analyzed memory B cell populations found in the peripheral blood upon XBB.1.5 S vaccination by measuring the frequency of XBB.1.5 RBD-reactive memory B cells which also bound to the Wuhan-Hu-1 RBD using flow cytometry. Only 5 of the 12 individuals profiled had memory B cells that recognized the XBB.1.5 RBD, but not the Wuhan-Hu-1 RBD, and these memory B cells were rare (0.4–13.3%) (Figure 1G, Figures S4–S5 and Table S1 in the Supplementary Appendix). Given that the vast majority of XBB.1.5 RBD-binding memory B cells also bound to the Wuhan-Hu-1 RBD, *de novo* elicitation of memory B cells is possible through variant-specific mRNA booster, as is the case here for XBB.1.5, but difficult to induce due to preferential recall of pre-existing Wuhan-Hu-1 memory B cells.

The lack of detectable plasma antibodies specific for XBB.1.5 S and the scarcity of memory B cells binding to the XBB.1.5 RBD, but not the Wuhan-Hu-1 RBD, indicate that the humoral immune responses elicited by XBB.1.5 S vaccination are dominated by recall of pre-existing memory B cells previously induced by Wuhan-Hu-1 S vaccination instead of inducing *de novo* responses against this new variant. These findings concur with observations made after Omicron BA.1, BA.2 and BA.5 breakthrough infections^11,13^ and with that made after the roll out of the bivalent Wuhan-Hu-1/BA.5 and Wuhan-Hu-1/BA.1 S vaccine boosters^12,15^. As SARS-CoV-2 S-specific memory B cells continue to evolve and increase in frequency several months post infection or vaccination^17–21^, future analysis at later time points will shed light on long-term immunological impact of imprinting. Collectively, our results indicate that the updated XBB.1.5 S vaccine elicit neutralizing antibodies against circulating variants and point to the persistence of immune imprinting which will need to be considered to guide the design of future vaccine boosters.

## Supplementary Material

1

## Figures and Tables

**Figure 1.**
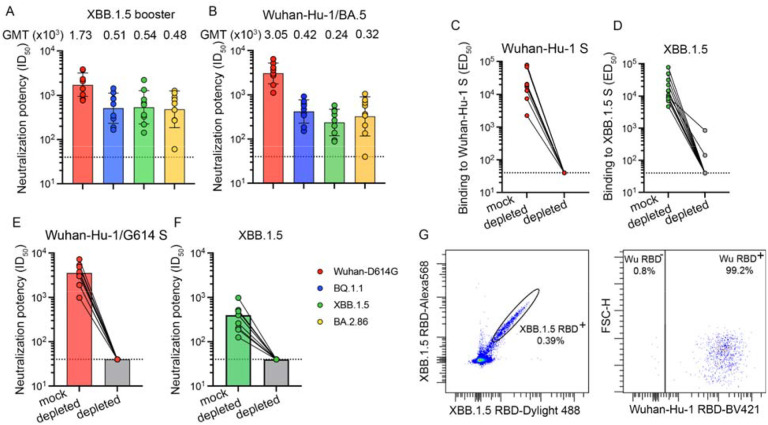
Immune imprinting dominates the immune response elicited upon XBB.1.5 S mRNA booster vaccination in humans. Panels A and B show plasma neutralizing antibody titers evaluated using a vesicular stomatitis virus (VSV) pseudotyped with the Wuhan-Hu-1 S harboring the D614G mutation, the BQ.1.1 mutations, the XBB.1.5 mutations or the BA.2.86 mutations using plasma obtained 7 to 13 days (mean: 9.7 days) after vaccination with the XBB.1.5 S mRNA booster (A) or 22 to 51 days (mean: 33 days) after vaccination with the bivalent Wuhan-Hu-1/BA.5 S mRNA booster. Geometric mean titers (GMTs) against each pseudovirus are indicated above the plot. Panels C and D show antibody binding titers (expressed as mean effective dilution 50%, ED_50_) against Wuhan-Hu-1 S (C) and XBB.1.5 S (D) before (left) and after (right) depletion of antibodies recognizing Wuhan-Hu-1 S, as determined by ELISA using XBB.1.5 S vaccinee plasma. Panels E and F show neutralizing antibody titers against and XBB.1.5 S (E) and Wuhan-Hu-1 S VSV (F) mock-depleted (left column) and after depletion (right column) of antibodies recognizing Wuhan-Hu-1 S. The dotted lines indicate the limit of detection of 1/40 for all assays. Data shown are from one representative (out of two) biological replicates with each point corresponding to the average of two technical replicates. Panel G shows the analysis of XBB.1.5 and Wuhan-Hu-1 RBD binding of memory B cells obtained from peripheral blood of individuals who received the XBB.1.5 S mRNA booster using flow cytometry.
