# Supplementary material for: Persistent immune imprinting after XBB.1.5 COVID vaccination in humans
Source: bioRxiv. 2023 Nov 30:2023.11.28.569129. Preprint. [Version 1] doi: 10.1101/2023.11.28.569129 (PMC10705481; doi:10.1101/2023.11.28.569129)
Supplement: 1 [file NIHPP2023.11.28.569129V1-supplement-1.pdf]

bioRxiv preprint doi: https://doi.org/10.1101/2023.11.28.569129; this version posted November 30, 2023. The copyright holder for this preprint (which was not certified by peer review) is the author/funder, who has granted bioRxiv a license to display the preprint in perpetuity. It is made available under aCC-BY 4.0 International license.

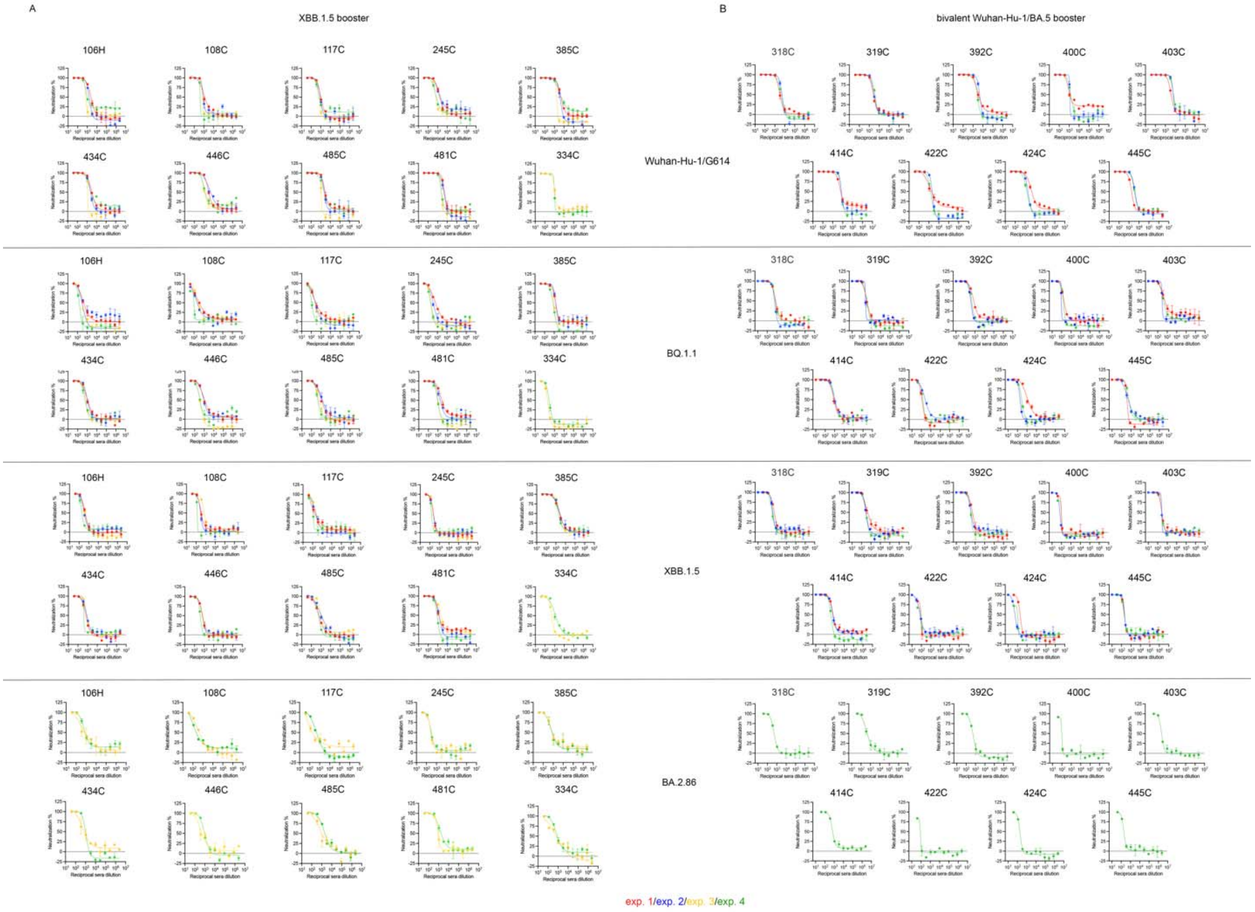

bioRxiv preprint doi: https://doi.org/10.1101/2023.11.28.569129; this version posted November 30, 2023. The copyright holder for this preprint (which was not certified by peer review) is the author/funder, who has granted bioRxiv a license to display the preprint in perpetuity. It is made available under aCC-BY 4.0 International license.

Figure S1. Plasma neutralizing antibody titers after vaccination with the XBB.1.5 S mRNA booster (A) and after the bivalent Wuhan-Hu-1/BA.5 S mRNA booster (B). Dose-response curves for all repeats are shown with each patient ID indicated on top of the graphs. Experiments 1 and 2 (exp. 1 and exp. 2) were performed using a first batch of VSV S pseudotyped viruses using VeroE6-TMPRSS2 cells puromycin resistant. Experiments 3 and 4 (exp. 3 and exp 4) were performed using a second batch of VSV S pseudotyped viruses using VeroE6-TMPRSS2 cells geneticin resistant for exp. 3 and VERO-TMPRSS2 cell puromycin resistant for exp. 4.

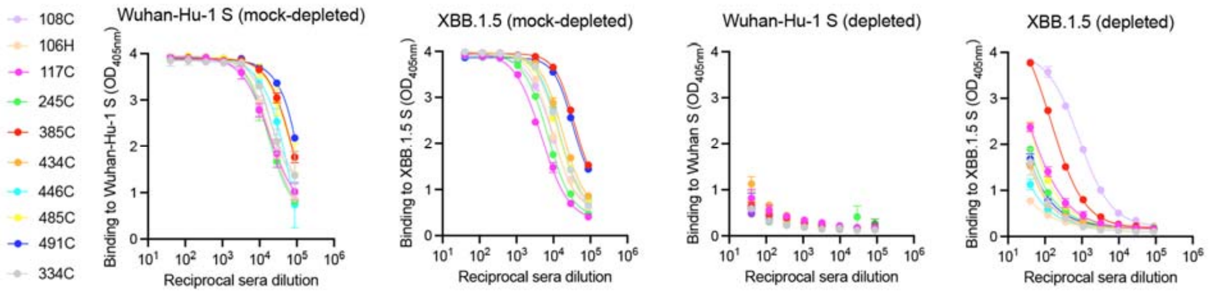

Figure S2. Plasma antibody binding titers against Wuhan-Hu-1 S and XBB.1.5 S after vaccination with the XBB.1.5 S mRNA booster in plasma samples mock-depleted and depleted of Wuhan-Hu-1 S-directed antibodies. Dose-response curves for one representative experiment are shown. The color key indicates patient IDs.

bioRxiv preprint doi: https://doi.org/10.1101/2023.11.28.569129; this version posted November 30, 2023. The copyright holder for this preprint (which was not certified by peer review) is the author/funder, who has granted bioRxiv a license to display the preprint in perpetuity. It is made available under aCC-BY 4.0 International license.

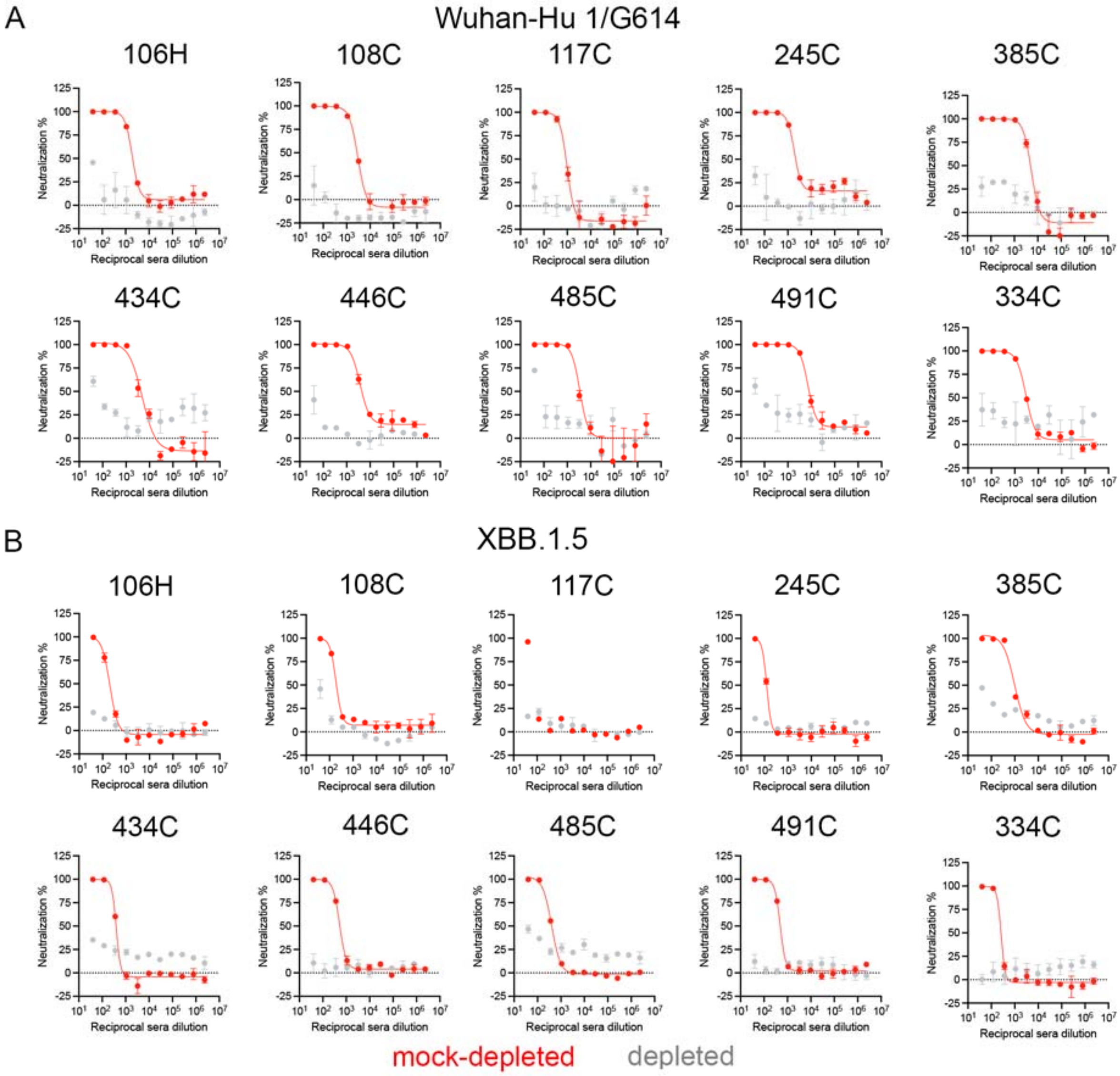

Figure S3. Plasma neutralizing antibody titers after vaccination with the XBB.1.5 S mRNA booster in plasma samples mock-depleted and depleted of Wuhan-Hu-1 S-directed antibodies. Dose-response curves for one representative experiment are shown. Patient IDs are indicated on top of each graph.

bioRxiv preprint doi: https://doi.org/10.1101/2023.11.28.569129; this version posted November 30, 2023. The copyright holder for this preprint (which was not certified by peer review) is the author/funder, who has granted bioRxiv a license to display the preprint in perpetuity. It is made available under aCC-BY 4.0 International license.

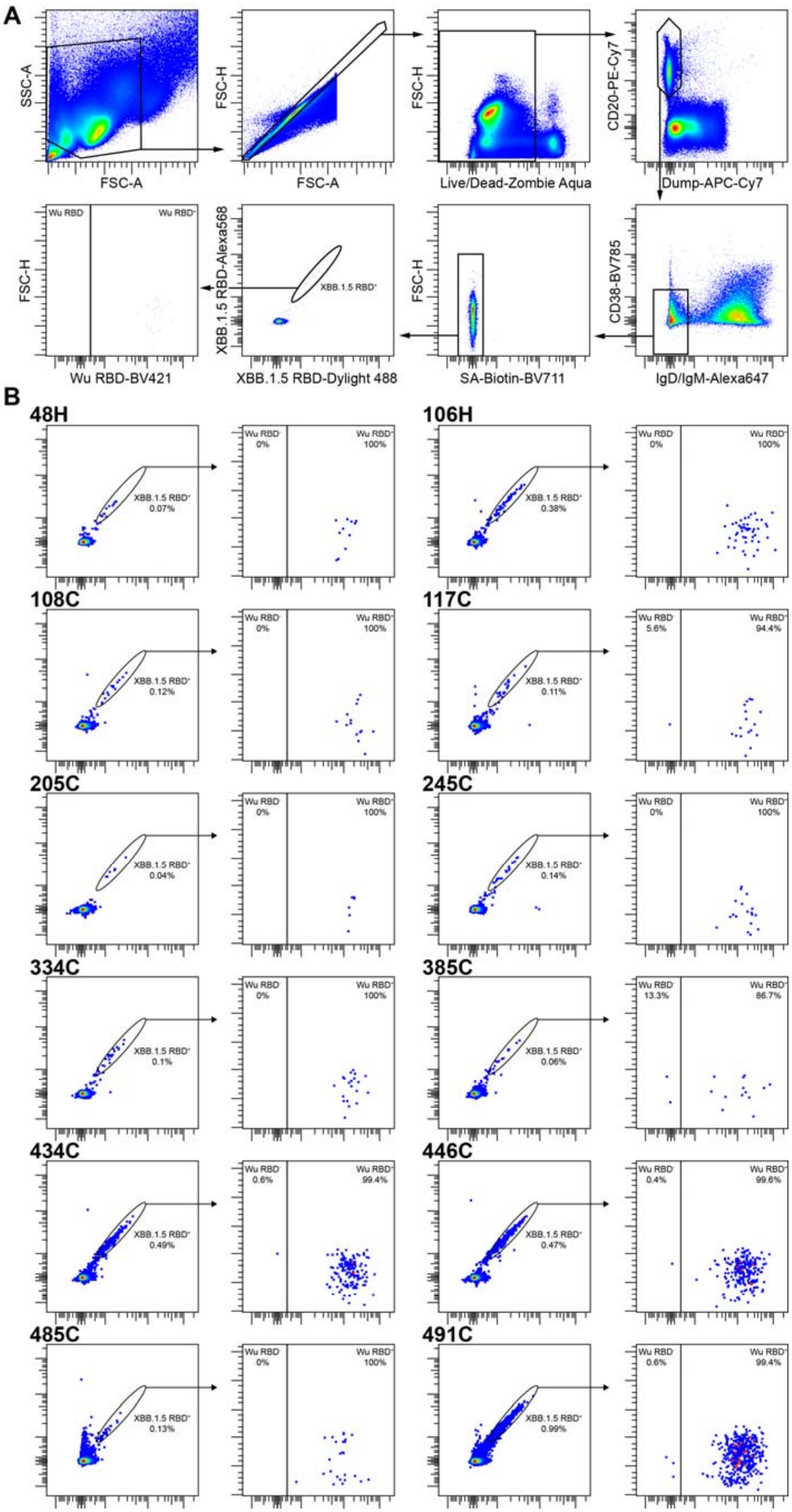

bioRxiv preprint doi: https://doi.org/10.1101/2023.11.28.569129; this version posted November 30, 2023. The copyright holder for this preprint (which was not certified by peer review) is the author/funder, who has granted bioRxiv a license to display the preprint in perpetuity. It is made available under aCC-BY 4.0 International license.

Figure S4. Flow cytometry analysis of memory B cells. Panel A shows the gating strategy to evaluate the cross-reactivity with the Wuhan-Hu-1 RBD of XBB.1.5 RBD+ memory B cells. Dump includes markers for CD3, CD8, CD14, and CD16. Panel B shows the gating of XBB.1.5 RBD-reactive memory B cells and subsequent Wuhan-Hu-1 RBD-binding of these memory B cells for each individual profiled.

Patient ID	Number of pre-Omicron SARS-CoV-2 infection (before 11/2021)	Number of post-Omicron SARS-CoV-2 infection (after 11/2021)	Number of Wuhan-Hu-1 vaccine doses	Number of bivalent Wuhan-Hu-1/BA.5 vaccine doses	Number of XBB.1.5 vaccine doses	Days between XBB.1.5 vaccine and blood draw
106H	0	0	3	1	1	7
108C	1	1	4	2	1	8
117C	1	0	4	1	1	10
245C	1	1	3	1	1	9
334C	0	1	3	1	1	13
385C	1	0	3	1	1	11
434C	0	1	4	1	1	11
446C	0	1	4	0	1	13
485C	0	1	4	1	1	11
491C	0	1	4	1	1	10

Table S1. Demographics information of the subjects in the XBB.1.5 vaccinee cohorts

bioRxiv preprint doi: https://doi.org/10.1101/2023.11.28.569129; this version posted November 30, 2023. The copyright holder for this preprint (which was not certified by peer review) is the author/funder, who has granted bioRxiv a license to display the preprint in perpetuity. It is made available under aCC-BY 4.0 International license.

Patient ID	Number of pre-Omicron SARS-CoV-2 infection (before 11/2021)	Number of post-Omicron SARS-CoV-2 infection (after 11/2021)	Number of Wuhan-Hu-1 vaccine doses	Number of bivalent Wuhan-Hu-1/BA.5 vaccine doses	Number of XBB.1.5 vaccine doses	Days between bivalent Wuhan-Hu-1/BA.5 vaccine and blood draw
318C	0	1	3	1	0	43
319C	0	1	3	1	0	29
392C	0	1	5	1	0	41
400C	0	1	3	1	0	23
403C	0	1	3	1	0	34
414C	0	1	3	1	0	52
422C	0	1	3	1	0	37
424C	0	1	3	1	0	30
445C	0	1	3	1	0	37

Table S2. Demographics information of the subjects in the Wuhan-Hu-1/BA.5 bivalent vaccinee cohorts

Supplementary Methods

Cell lines

Cell lines used in this study were obtained from JCRB-Cell Bank (VeroE6-TMPRSS2, geneticin resistant) and ThermoFisher Scientific (Expi293F™ cells). Cells were cultivated at 37°C, in an atmosphere of 5 % CO_2 and with 130 rpm of agitation for suspension cells. None of the cell lines

bioRxiv preprint doi: https://doi.org/10.1101/2023.11.28.569129; this version posted November 30, 2023. The copyright holder for this preprint (which was not certified by peer review) is the author/funder, who has granted bioRxiv a license to display the preprint in perpetuity. It is made available under aCC-BY 4.0 International license.

used were routinely tested for mycoplasma contamination. The VeroE6-TMPRSS2 puromycin resistant cells were previously described[22].

Plasmids

Plasmids encoding Wuhan-Hu-1 S Hexapro ectodomain (residues 1-1208) and XBB.1.5 S Hexapro ectodomain residues (residues 1-1203) were synthesized into pCDNA 3.1 (-) and pcDNA 3.1 (+), respectively. Both genes were synthesized by Genscript and harbor the HexaPro mutations[23], a wildtype signal peptide, a furin cleavage site mutated $_{685}$RSV$_{687}$ to $_{685}$SSV$_{687}$, an avi-tag, and an octa-his tag for affinity purification.

Constructs for membrane-anchored S glycoproteins from SARS-CoV-2 Wuhan-D614G, BQ.1.1, XBB.1.5 and BA.2.86 contain the wild-type signal peptide and a 21-amino acid C-terminal deletion[24] and were all synthesized and cloned into HDM vector. Genes were synthesized by Genscript with codon optimized for expression in mammalian cells, without tag and cloned in frame with a Kozak's sequence to direct translation.

Plasmids encoding the SARS-CoV-2 Wuhan-Hu-1 and the XBB.1.5 RBDs were previously described elsewhere[12,25]. The SARS-CoV-2 Wuhan-Hu-1 RBD construct contains an N-terminal mu-phosphatase signal peptide and C-terminal octa-histidine tag followed by an avi-tag. The XBB.1.5 RBD construct contains an N-terminal BM40 signal peptide and a C-terminal octa-histidine tag followed by an avi-tag. The boundaries of both the Wu and XBB.1.5 RBD constructs are N-$_{328}$RFPN$_{331}$ and C-$_{528}$KKST$_{532}$.

Recombinant protein production

SARS-CoV-2 Wuhan-Hu-1 and XBB.1.5 HexaPro S ectodomains were expressed in Expi293 cells at 37°C and 8% CO_2. Cells were transfected with the corresponding plasmids using Expifectamine following the manufacturer's instructions. Four days post-transfection, supernatants were clarified by centrifugation at 4121g for 30 minutes, supplemented with 25 mM phosphate pH 8.0, 300 mM NaCl, and 0.5 mM phenylmethylsulfonyl fluoride (PMSF). Supernatant was then 0.22µm vacuum filtered and passed through 1 mL His trap HP or Excel column (Cytiva) previously equilibrated in 25 mM phosphate pH 8.0, 300 mM NaCl. S proteins were eluted using a buffer identical to the binding buffer with the addition of 300 mM imidazole. Fractions containing the proteins were pooled and buffer exchanged to 50 mM Tris-HCl pH 8.0, 150 mM NaCl and stored at 4°C or immediately used.

The SARS-CoV-2 Wu and XBB.1.5 RBD proteins were expressed and purified as described above. Following buffer exchange, the purified RBDs were biotinylated using the BirA biotin-protein ligase reaction kit (Avidity). The biotinylated proteins were passed, washed, and eluted again on the same affinity column, concentrated and ran over a Superdex200 increase 10/300 size-exclusion column (Cytiva). Fractions corresponding to monomeric and monodisperse RBDs were collected, flash frozen, and stored at -80°C until use.

Plasma antibody depletion

Invitrogen His-Tag Dynabeads (ThermoFisher 10104D) were used for depletion of plasma samples from antibodies recognizing the Wuhan-Hu-1 S trimer, as previously described[9] with

bioRxiv preprint doi: https://doi.org/10.1101/2023.11.28.569129; this version posted November 30, 2023. The copyright holder for this preprint (which was not certified by peer review) is the author/funder, who has granted bioRxiv a license to display the preprint in perpetuity. It is made available under aCC-BY 4.0 International license.

some modifications. Vortexed beads were incubated at room temperature on an Invitrogen DynaMag-2 Magnet (ThermoFisher 12–321-D) for two minutes to allow beads to separate for the liquid phase. Supernatant was discarded and beads were washed one time with TBS-T and divided in two tubes. After a two-minute incubation on the magnet, TBS-T supernatants were discarded and one set of beads was incubated with 4 mg of purified his-tagged SARS-CoV-2 Wuhan-Hu-1 S ectodomain trimer (Wuhan-Hu-1 S depletion) and the other set was incubated with TBS-T alone (mock depletion) with gentle rotation for 1 h at room temperature. Supernatants were discarded using the magnet and beads were washed three times with TBS-T. Subsequently, 20 µl of each of the plasma samples were incubated with 80 µl of S-loaded beads or mock-loaded beads for 1 h at 37°C during which time they were mixed every 15 min. Plasma samples were recovered using the magnet to separate the beads and used for neutralization assays.

VSV pseudotyped virus production

Vesicular stomatitis virus (VSV) were pseudotyped with the SARS-CoV-2 S proteins corresponding to Wuhan-D614G, BQ.1.1, XBB.1.5 and BA.2.86 variants following a previously described protocol[26]. Briefly, HEK293T cells seeded in poly-D-lysine coated 10-cm dishes in DMEM supplemented with 10% FBS and 1% PenStrep were transfected with a mixture of 24 µg of the corresponding plasmids, 60 µl Lipofectamine 2000 (Life Technologies) in 5 ml of Opti-MEM, following the manufacturer's instructions. After 5 h at 37°C, 5 ml of DMEM supplemented with 20% FBS and 2% PenStrep were added. The next day, cells were washed three times with DMEM and were transduced with VSVΔG-luc[27]. After 2 h, the virus inoculum was removed and cells were washed five times with DMEM prior to the addition of DMEM supplemented with anti-VSV-G antibody [Il-mouse hybridoma supernatant diluted 1 to 25 (v/v), from CRL-2700, ATCC] to minimize parental background. After 18-24 h, supernatants containing pseudotyped VSV were harvested, centrifuged at 2,000 x g for 5 min to remove cellular debris, filtered with a 0.45 µm membrane, concentrated 10 times using a 30 kDa cut off membrane (Amicon), aliquoted, and frozen at -80°C.

Pseudotyped VSV neutralization

VeroE6-TMPRSS2 cells were seeded into coated clear bottom white walled 96-well plates at 40,000 cells/well and cultured overnight at 37°C. Eleven 3-fold serial dilutions of each plasma sample were prepared in DMEM. Pseudotyped VSV viruses, diluted 1 to 20 in DMEM containing anti-VSV-G antibody, were added 1:1 (v/v) to each plasma sample dilution and mixtures of 50 µl volume were incubated for 45-60 min at 37°C. VeroE6-TMPRSS2 cells were washed three times with DMEM and 40 µL of the mixture containing pseudotyped virus and plasma samples were added. Two hours later, 40 µL of DMEM were added to the cells. After 17-20 h, 70 µL of One-Glo-EX substrate (Promega) were added to each well and incubated on a plate shaker in the dark at 37°C. After 5-15 min incubation, plates were read on a Biotek Neo2 plate reader. Measurements were done in duplicate or triplicate with at least two biological replicates. Relative luciferase units were plotted and normalized in Prism (GraphPad): cells without pseudotyped virus added were defined as 0 % infection or 100 % neutralization, and cells with virus only (no plasma) were defined as 100 % infection or 0 % neutralization.

bioRxiv preprint doi: https://doi.org/10.1101/2023.11.28.569129; this version posted November 30, 2023. The copyright holder for this preprint (which was not certified by peer review) is the author/funder, who has granted bioRxiv a license to display the preprint in perpetuity. It is made available under aCC-BY 4.0 International license.

Enzyme-linked immunosorbent assays (ELISA)

Analysis of plasma binding antibodies for samples mock-depleted or depleted of antibodies binding to the Wuhan-Hu-1 S ectodomain trimer was performed using ELISAs. Briefly, clear flat bottom Immuno Nonsterile 384-well plates (Thermo Scientific) were coated overnight at room temperature with 30 µl of Wuhan-Hu-1 S or XBB.1.5 S prepared at 3 µg/ml in PBS (137 mM of NaCl, 2.7 mM of KCl, 10 mM of Na2HPO4, and 1.8 mM of KH2PO4, pH 7.2). The next day, plates were blocked with Blocker™ Casein (Thermo Scientific) and subsequently incubated with serial dilutions of plasma samples for 1 h at 37°C. After four washing steps with TBS-T, goat anti-human IgG-Fc secondary antibody conjugated to HRP (Invitrogen A18817, diluted 1/500) was added and incubated for 1 h at 37°C. Plates were washed four times with TBS-T and KPL SureBlue Reserve™ TMB Microwell Peroxidase Substrate (VWR) was added. After 2 min incubation, 1N HCl was added and absorbance at 405 nm was measured using a Biotek Neo2 plate reader. Data were plotted using GraphPad Prism 9.1.0.

Flow cytometry analysis of SARS-CoV-2 RBD-reactive memory B cells

To define specific B cell populations reactive with the XBB.1.5 and the Wuhan-Hu-1 RBDs, RBD–streptavidin tetramers conjugated to fluorophores were generated by incubating biotinylated RBDs with streptavidin at a 4:1 molar ratio for 30 min at 4 °C. Excess of free biotin was then added to the reaction to bind any unconjugated sites in the streptavidin tetramers. The RBD-streptavidin tetramers were washed once with PBS and concentrated with a 100-kDa cut-off centrifugal concentrator (Amicon). An additional streptavidin tetramer conjugated to biotin only was generated and included in the staining as decoy.

Approximately 5 to 15 million PMBCs were collected 7 to 13 days post-vaccination for individuals who received an XBB.1.5 S mRNA vaccine booster. Cells were collected by centrifugation at 400g for 2 mins at 4°C, washed twice with PBS and stained with Zombie Aqua dye (Biolegend; Catalog No. 423101; diluted 1:100 in PBS) at room temperature. After 30 min incubation, cells were washed twice with PBS and stained with antibodies for CD20-PECy7 (BD; Catalog No. 335793), CD3-Alexa eFluor780 (Thermo Fisher; Catalog No. 47-0037-41), CD8-Alexa eFluor780 (Thermo Fisher; Catalog No. 47-0086-42), CD14-Alexa eFluor780 (Thermo Fisher; Catalog No. 47-0149-42), CD16-Alexa eFluor780 (Thermo Fisher; Catalog No. 47-0168-41), IgM-Alexa Fluor 647 (BioLegend; Catalog No. 314535), IgD-Alexa Fluor 647 (BioLegend; Catalog No. 348227), and CD38-Brilliant Violet 785 (BioLegend; Catalog No. 303529), all diluted 1:200 in Brilliant Stain Buffer (BD; Catalog No. 563794), along with the RBD-streptavidin tetramers for 30 min at 4°C. Cells were washed three times, resuspended in PBS, and passed through a 35-µm filter before being examined on a BD FACSymphony A3 for acquisition and FlowJo 10.8.1 for analysis. Gates for identifying the XBB.1.5 RBD double-positive population as well as the subsequent Wuhan-Hu-1 RBD-positive and Wuhan-Hu-1 RBD-negative populations were drawn based on staining of fluorescent minus one controls.
